# Thermal Programming of Commercially Available Orthodontic NiTi Archwires

**DOI:** 10.3390/ma16103683

**Published:** 2023-05-11

**Authors:** Andrea Wichelhaus, Amelie Mehnert, Thomas Stocker, Uwe Baumert, Matthias Mertmann, Hisham Sabbagh, Corinna L. Seidel

**Affiliations:** Department of Orthodontics and Dentofacial Orthopaedics, University Hospital, LMU Munich, Goethe-Strasse 70, 80336 Munich, Germany; amelie.mehnert@med.uni-muenchen.de (A.M.); th.stocker@med.uni-muenchen.de (T.S.); uwe.baumert@med.uni-muenchen.de (U.B.); matthias.mertmann.extern@med.uni-muenchen.de (M.M.); hisham.sabbagh@med.uni-muenchen.de (H.S.); corinna.seidel@med.uni-muenchen.de (C.L.S.)

**Keywords:** Nickel-Titanium, NiTi archwires, thermal shape adjustment, heat treatment, annealing temperature, annealing duration, orthodontic materials, orthodontic wires, superelasticity

## Abstract

The shape of superelastic Nickel-Titanium (NiTi) archwires can be adjusted with thermal treatments using devices such as the Memory-Maker^TM^ (Forestadent), which potentially affects their mechanical properties. The effect of such treatments on these mechanical properties was simulated by means of a laboratory furnace. Fourteen commercially available NiTi wires (0.018″ × 0.025″) were selected from the manufacturers American Orthodontics, Dentaurum, Forestadent, GAC, Ormco, Rocky Mountain Orthodontics and 3M Unitek. Specimens were heat treated using different combinations of annealing duration (1/5/10 min) and annealing temperature (250–800 °C) and investigated using angle measurements and three-point bending tests. Complete shape adaptation was found at distinct annealing durations/temperatures for each wire ranging between ~650–750 °C (1 min), ~550–700 °C (5 min) and ~450–650 °C (10 min), followed by a loss of superelastic properties shortly afterwards at ~750 °C (1 min), ~600–650 °C (5 min) and ~550–600 °C (10 min). Wire-specific working ranges (complete shaping without loss of superelasticity) were defined and a numerical score (e.g., stable forces) was developed for the three-point bending test. Overall, the wires Titanol Superelastic (Forestadent), Tensic (Dentaurum), FLI CuNiTi^27^ (Rocky Mountain Orthodontics) and Nitinol Classic (3M Unitek) proved to be the most user-friendly. Thermal shape adjustment requires wire-specific working ranges to allow complete shape acceptance and high scores in bending test performance to ensure permanence of the superelastic behaviour.

## 1. Introduction

Nickel-Titanium (NiTi) alloys have a wide range of applications in orthodontics, above all as archwires in treatment within fixed appliances. Superelastic NiTi archwires are used for initial aligning and tooth irregularity correction and represent a viable addition to conventional steel archwires [1], which can easily be bent. However, they fatigue quickly due to frequent reactivations and force control is more difficult because of their high elastic modulus, with a latent risk for root resorptions [2]. This risk can be minimized using superelastic NiTi archwires, since small and constant forces over long displacements are ensured by the horizontal force plateau of the unloading curve of the material [2,3], which is based on reverse martensitic transformation. The risk for root resorptions can also be minimized by applying individual forces with regard to tooth root surfaces, i.e., lower forces should be applied on front teeth, while higher forces can be used for tooth movement of premolars and molars. To optimize those force levels, multi-force orthodontic NiTi archwires with reduced forces in the anterior region and greater forces in the posterior region of the archwire were developed, and were shown to maintain their superelastic properties and stable force values after usage of over 8 weeks [4]. Notably, new technologies, such as the ageing of NiTi archwires, are used to increase flexibility and reduce the force levels necessary for the aligning of extremely malpositioned teeth [5]. In contrast to steel arches, the bending of NiTi archwires is difficult due to their high elastic strain limit, however, shape adaptation is highly important in different stages of treatment with fixed appliances, e.g., to maintain the individual intercanine distance by compression or transversal expansion of the conventional NiTi wires necessary for stable treatment results [6], or to initiate deep bite treatment using sweep [7]. The shape of superelastic NiTi archwires, as well as the position of the unloading plateau, can be clinically adjusted by cold forming or heat treatment using a furnace [8] or an direct electric resistance heater, e.g., the Memory-Maker^TM^ (Forestadent, Pforzheim, Germany) [7].When using the electrical current a segmental heating between the pliers is possible, while the heating depends on the magnitude and duration of the current flow, the cross-section and the length of the annealed wire segment as well as the point of application of the pliers, as the section between the pliers does not exhibit uniform temperatures. Since temperature control on fine wires is technically very challenging, the manufacturer refers to the golden yellow colouring of the wire’s oxide surface when adjusting the shape, while blue coloration of the wire’s oxide surface indicates overheating and loss of superelastic properties, resulting in an unusable wire. A working range of 400–600 °C is recommended by the device manufacturer during programming of the wire, however, temperatures between 500–600 °C were shown to decrease the force of the unloading curve and temperatures above 600 °C resulted in a loss of superelastic properties [9]. Overall, wire programming with an electrical current is hindered due to poor temperature control and unpredictable permanent deformation of the wire. In contrast to electrical current, total heating of the wire is inevitable, and the use of tooling is necessary to program the wire shape with heat treatment when using a furnace. The temperature in the furnace and the tool can be tracked by a temperature indicator. However, so far the temperature and exposure time required to achieve a desired shape was not predictable [8]. It is therefore desirable to determine both the effects of heat treatment duration and the absolute value of the heat treatment temperature on the extent of shape change and mechanical properties, to ensure complete shape adaptation without loss of superelasticity and enable the further development of thermal heat treatment of NiTi archwires in orthodontic practice.

Therefore, the aim of this study was to thermally adjust commercial NiTi archwires into a defined shape by varying annealing durations and temperatures using heat treatment with a laboratory furnace and a shape setting tool. Moreover, the study aimed to analyse the relationships between complete shape acceptance and annealing duration and temperature, as well as the resulting impact on material properties. The overarching goal was to identify the ideal wire, which can be reliably adjusted in its shape while at the same time largely retaining its material properties, to increase control of shape-setting in the long term.

## 2. Materials and Methods

In the present study, NiTi wires were subjected to a reshaping heat treatment in a ceramic press furnace (AUSTROMAT D4 oral design, DEKEMA, Freilassing, Germany) to simulate the possible side effects of thermal treatment on the mechanical behaviour of the alloy. Fourteen commercially available superelastic NiTi wires (0.018″ × 0.025″) were selected and investigated (Table 1).

**Figure 1:**
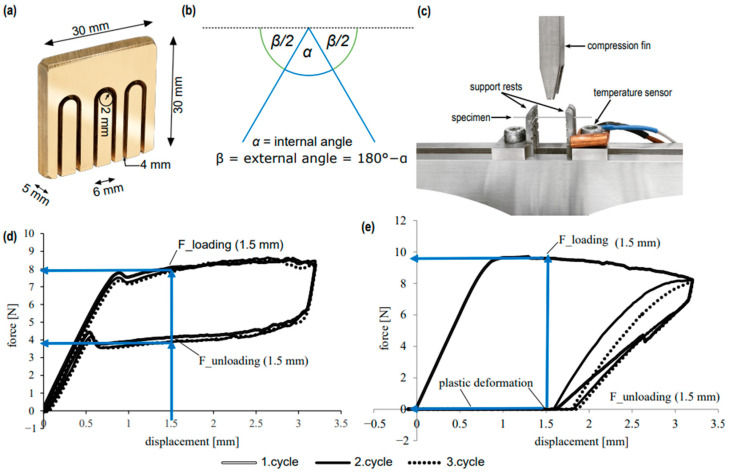
Visualisation of materials and methods. (**a**) Brass block with milled u-groove for wire shaping. (**b**) Measurement of the internal and external angle after removal post heat treatment. (**c**) 3-point bending test: positioning of the specimen on the support rests. (**d**) Three test cycles of a heat-treated NiTi wire in the force [N]-displacement [mm] diagram with F_loading/F_unloading (1.5 mm). (**e**) Three test cycles of a wire in the force [N]-displacement [mm] diagram with F_loading/F_unloading (1.5 mm) showing poor superelastic performance after heat treatment.

The straight segments of the samples (~50 mm) were cut and placed in the mould (30 mm × 30 mm × 5 mm) for shape setting by heat treatment (Figure 1a). Three u-shaped grooves were milled into each brass block in a pantograph engraving machine (Kuhlmann, Bad Lauterberg, Germany) with the following dimensions:

- Length of the straight u-shaped legs: 20 mm;
- Distance between the two u-shaped legs: 6 mm;
- Arch radius of the u-shaped grooves: 2 mm;
- Engraving depth: 4 mm.

To investigate the impact of thermal treatment, specimens from each specimen group were tested for each temperature–annealing combination (250–800 °C with increments of 50 °C; one, five or ten minutes). Once the set annealing temperature was reached, the furnace that ensured constant temperatures (±1 °C) opened automatically, and the brass block with the clamped wire specimens was placed on the firebrick in the furnace. After cooling to room temperature, the samples were removed and examined using angle and 3-point bending tests.

Angle test: in order to determine the extent of shape adaptation, the specimens were optically scanned (HP Officejet 4500 G510n-z, Hewlett-Packard, Houston, TX, USA) at an optical resolution of 2400 × 4800 PPI and the angle between legs was determined using the Image J program [10]. The external angle β was used in the data evaluation and calculated using the formula: β = 180° − α (internal angle, maximum of 180°) (Figure 1b). When angle measurement revealed an external angle β of 180 ± 3°, it was defined as complete shape adaptation. Angle β << 180° indicated that specimens did not assume the new shape at all. For each sample group (annealing temperature/annealing time), the mean value including standard deviation was calculated and graphically represented in the form of annealing temperature [°C]-external angle [°]-diagrams.

Three-point bending test: the Zwick Roell Z5.0 materials testing machine (Zwick Roell, Ulm, Germany) and the associated testXpert^®^ II testing software (software version number 3.41) were used to examine material properties. Two untreated and four heat-treated specimens from each specimen group (annealing temperature/annealing duration) were examined to determine their force-deformation behaviour. According to the DIN EN ISO 15841:2013 standard for non-linear elastic wires type 2, the tests were performed at constant temperatures of 36 °C (±1 °C) ensured by the Domotherm Easy digital thermometer (Uebe Medical GmbH, Wertheim-Reicholzheim, Germany). Before starting the measurement, the specimen was positioned with its flat side on the two support rests (distance interjacently: 10 mm) (Figure 1c). During the measurement, the centrally positioned compression fin (Figure 1c) performed a total deflection of the wire specimen by 3.1 mm at a speed of 7.0 mm/min, followed by a relief at a constant speed and the restart of the load/unload cycles (per specimen: n = 3). The testing software TestXpert II (Zwick Roell) recorded the parameters test time t [s], displacement s [mm] and force F [N] (Figure 1d). In order to be able to compare the specimens with each other, the forces F_loading or F_unloading [N] of the 1st test cycle at a deflection of s = 1.5 mm were determined for the loading and unloading curves, respectively, as a function of the two parameters annealing temperature and duration (Figure 1e). These data points were chosen because they were located in the horizontal force plateau of the loading and unloading curves for all manufacturers. The 2nd and 3rd cycles were each not analysed further, as they often differed only slightly from the first cycle. The loss of superelastic properties could be evaluated from the *x*-axis intercept of the 1st cycle curves in the force–displacement diagram. Characteristic of these specimens was the failure to spring back to their original shape after the first loading and unloading cycle: permanent (plastic) deformation (Figure 1e).

A so-called temperature working range was defined, within which a complete shape acceptance (β = 180 ± 3° of the wire without loss of superelastic properties occurred. A newly designed evaluation scale (“score”) (Table 2) was defined, which was intended to express the quality of a superelastic wire, the change in mechanical properties and the sensitivity to thermal treatment. This score takes the complexity of the measured curves, as well as the clinical relevance of the related wire properties, into consideration.

A low score value meant that a wire underwent a major microstructural change due to heat treatment and was therefore sensitive to shape adjustments and the given parameters. A high score value indicated a wire with low thermal susceptibility and very little change in its property profile after heat treatment. An ideal type of wire with a high score (maximum score 3 × 38 = 114 points) had the following characteristics:

- A low loading and unloading force in the upper and lower plateau level to minimize periodontal damage.
- Stable force values up to high annealing temperature ranges. This expresses that a wire was insensitive to heat treatments in terms of its mechanical properties.
- A force drop with a low inclination (not steep) after high annealing temperatures or annealing times, which means that the wire’s properties do not change spontaneously once a critical temperature is reached.
- A monotonic curve with few fluctuations, as any change in force could lead to inadequate orthodontic results.

Descriptive statistics were generated using Microsoft Excel 2016 (Microsoft Corporation, Redmond, WA, USA). The mean values and standard deviations (SD) were calculated from each of the six (external angle) and four (3-point bend test) measurements of a product–annealing and time–annealing temperature combination. The graphical representation of the results was done as a function of the annealing temperature.

## 3. Results

### 3.1. Angle Measurements

**Figure 2:**
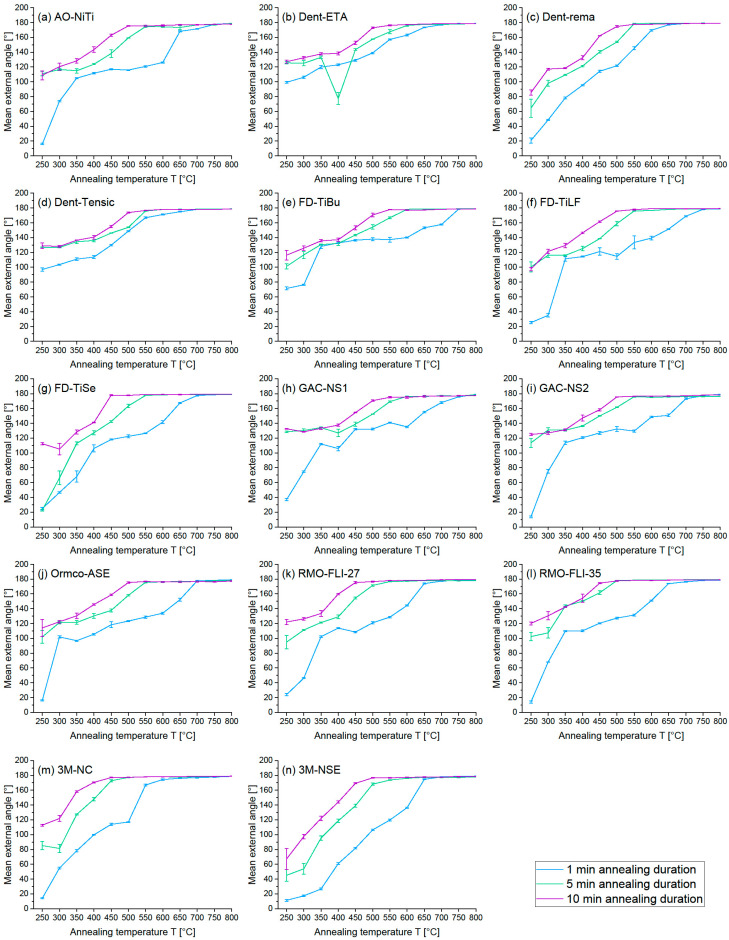
The measured external angle [°]-annealing temperature T [°C] diagram with SD (black) is given for all measured NiTi wires and three different annealing durations: (**a**) AO-NiTi, (**b**) Dent-ETA, (**c**) Dent-rema, (**d**) Dent-Tensic, (**e**) FD-TiBu, (**f**) FD-TiLF, (**g**) FD-TiSe, (**h**) GAC-NS1, (**i**) GAC-NS2, (**j**) Ormco-ASE, (**k**) RMO-FL127, (**l**) RMO-FL135, (**m**) 3M-NC, (**n**) 3M-NSE.

The graphical representation of the results of the angle measurement was in the form of external angle [°] -annealing temperature T [°C] diagrams for all wires (complete shape adaptation: external angle β = 180° ± 3°) (Figure 2).

Regarding the wires from American Orthodontics, Nickel Titanium (AO-NiTi) wires attained complete shaping at 750 °C (1 min), 700 °C (5 min) and at 650 °C (10 min); 5 and 10 min annealing durations resulted in similar curves and 1 min annealing duration showed moderate form changes at 250 °C, with a flat curve between 350 °C and 600 °C (Figure 2a).

Considering the wires from Dentaurum, Equire Thermo-Active (Dent-ETA) reached complete shaping at 700 °C (1 min), 650 °C (5 min) and at 600 °C (10 min), rematitan ‘LITE’ (Dent-rema) at 650 °C (1 min) and 550 °C (5 and 10 min) and Tensic (Dent-Tensic) at 700 °C (1 min), 600 °C (5 min) and at 550 °C (10 min) (Figure 2b–d). Contrary to AO-NiTi, moderate temperatures resulted in marked shaping for 1 min annealing duration for Dent-ETA and Dent-Tensic, while Dent-rema presented only slight shaping using low annealing temperatures.

Regarding the wires from Forestadent, Titanol Budget (FD-TiBu) and Titanol Low Force (FD-TiLF) wires achieved complete shaping at 750 °C (1 min), 600 °C (5 min) and at 550 °C (10 min), while Titanol Superelastic (FD-TiSe) wires already attained complete shaping at 700 °C (1 min), 550 °C (5 min) and at 450 °C (10 min) (Figure 2e–g). FD-TiBu and FD-TiLF presented similar curve progression for 5 and 10 min annealing durations. Notably, FD-TiBu presented severe shaping at 250 °C (5 and 10 min) and at 350 °C (1 min), while FD-TiSe showed severe shaping at 250 °C (10 min) but only slight form changes at 250 °C (1 and 5 min). Considering 1 min annealing durations, Forestadent wires presented a force plateau between 350 °C and 600 °C (FD-TiBu), as well as relatively stable force levels between 350 °C and 500 °C (FD-TiLF) and between 450 °C and 550 °C (FD-TiSe).

Considering the wires from GAC, Neo Sentalloy 02-526-652 (GAC-NS1) wires attained complete shaping at 750 °C (1 min), 600 °C (5 min) and at 550 °C (10 min) and Neo Sentalloy 02-523-653 (GAC-NS2) achieved complete shaping at 750 °C (1 min), 550 °C (5 min) and at 500 °C (10 min) (Figure 2h,i). Both GAC-NS1 and GAC-NS2 showed severe shaping at 250 °C (5 and 10 min) but only minor deformation at 250 °C (1 min).

Considering 1 min annealing durations, both GAC showed only slight shaping between 450 °C and 600 °C (GAC-NS1) and between 350 °C and 550 °C (GAC-NS2).

As regards the wires from Ormco, Align SE200 LM NiTi (Ormco-ASE) wires reached complete shaping at 700 °C (1 min) and at 650 °C (5 and 10 min) and presented only minor shaping at 250 °C (1 min), with a flat curve between 450 °C and 600 °C (Figure 2j).

Regarding the wires from RMO, FLI CuNiTi^27^ (RMO-FLI27) achieved complete shaping at 700 °C (1 min), 550 °C (5 min) and at 500 °C (10 min) and FLI CuNiTi^35^ (RMO-FLI35) wires at 750 °C (1 min) and at 500 °C (5 and 10 min) (Figure 2k,l). While 5 and 10 min annealing durations resulted in similar curves with severe shaping at 250 °C for RMO-FLI27FLI35, 1 min annealing showed a considerably smaller effect on shaping for both wires, with a plateau without deformation between 350 °C and 550 °C (RMO-FLI35).

The wires from 3M Unitek, Nitinol Classic (AM-NC), reached complete shaping at 650 °C (1 min), 500 °C (5 min) and at 450 °C (10 min) and Nitinol SuperElastic (AM-NSE) at 700 °C (1 min), 650 °C (5 min) and at 500 °C (10 min) (Figure 2m,n). In contrast to AM-NC, AM-NSE showed only slight form changes using low annealing temperatures and similar curve progression for all annealing temperatures.

### 3.2. Results of the Three-Point Bending Tests

**Figure 3:**
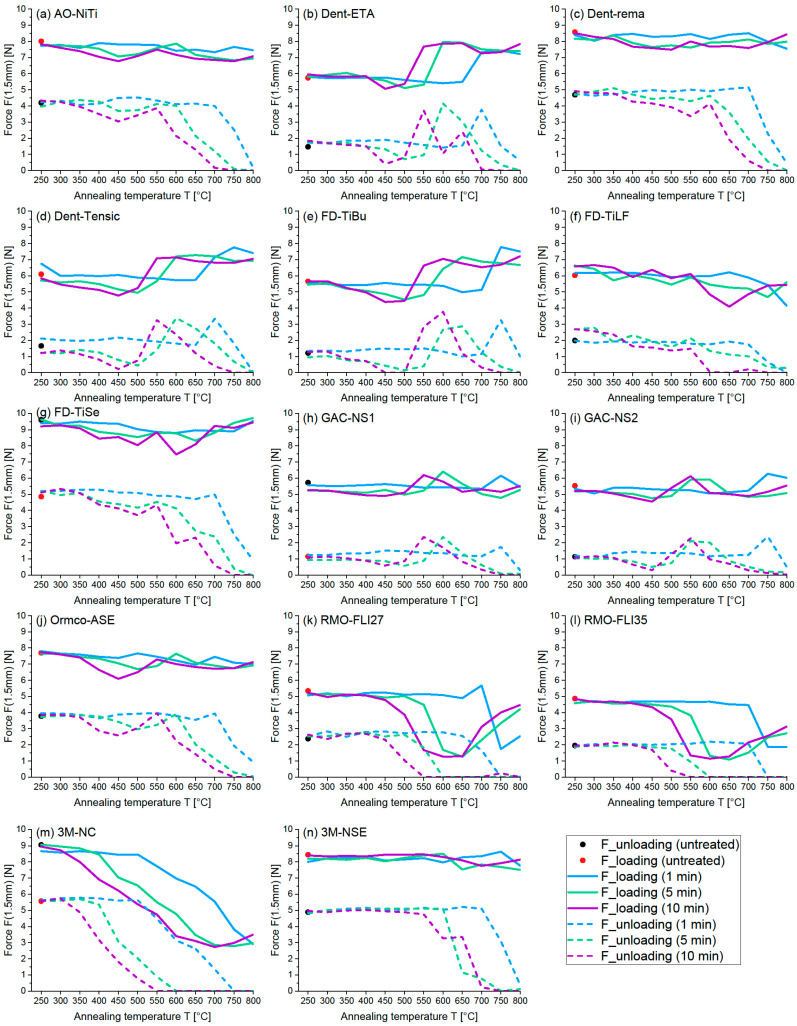
Force vs. annealing temperature T [°C] diagrams are given for all measured NiTi wires at three different annealing durations (1 min = blue; 5 min = green; 10 min = purple; black/red dot = loading and unloading forces (1.5 mm) of untreated reference samples): (**a**) AO-NiTi, (**b**) Dent-ETA, (**c**) Dent-rema, (**d**) Dent-Tensic, (**e**) FD-TiBu, (**f**) FD-TiLF, (**g**) FD-TiSe, (**h**) GAC-NS1, (**i**) GAC-NS2, (**j**) Ormco-ASE, (**k**) RMO-FL127, (**l**) RMO-FL135, (**m**) 3M-NC, (**n**) 3M-NSE.

Due to the complexity and great variety of curve progressions, the results of the three-point bending test were analysed using the force F [N] at a deflection of 1.5 mm for the loading as well as the unloading curves (Figure 3).

The AO-NiTi wires, presenting mean loading and unloading forces of 8.01 N and 4.2 N in an untreated condition, were insensitive to heat and relatively stable in shape regarding the loading curve. Tendentially, longer annealing durations resulted in reduced force levels. A loss of superelastic properties was found at annealing temperatures of 700 °C (1 min), 600 °C (5 min) and 550 °C (10 min), resulting in a continuous reduction in force (Figure 3a).

Considering the Dentaurum wires, the following mean forces were found for untreated Dent-ETA/-rema and -Tensic: F_loading = 5.74 N/8.58 N/6 N and F_unloading = 1.47 N/4.69 N/1.66 N (Figure 3b–d). Both Dent-ETA and Dent-Tensic wires presented an increase of force prior to the loss of superelastic properties. This force peak was followed by a remaining high force level regarding the loading curve, as well as a severe and finally complete loss of force regarding the unloading curve. Dent-rema wires were characterized by relatively stable force levels of the loading curve, regardless of annealing duration and annealing temperature. The unloading curve showed remaining force levels until loss of superelasticity resulting in a severe force loss afterwards. All Dentaurum wires (Dent-ETA/-rema/-Tensic) showed a loss of superelastic properties at 700 °C regarding 1 min and at 600 °C regarding 5 min annealing duration. However, differing annealing temperatures led to a loss of superelasticity regarding 10 min annealing duration: Dent-rema at 600 °C and Dent-ETA/-Tensic at 550 °C.

Concerning the Forestadent wires, untreated FD-TiBu/-TiLF and -TiSe showed the following mean forces: F_loading = 5.65 N/6.03 N/9.60 N, F_unloading = 1.22 N/1.99 N/4.85 N (Figure 3e–g). FD-TiBu and FD-TiLF were relatively insensitive to heat and stable in form regarding 1 min annealing temperature up to 700 °C and 650 °C, respectively, while FD-TiBu presented a strong force increase between 700*–*750 °C prior to loss of superelasticity and FD-TiLF showed a reduction of force at 650 °C. The 5 and 10 min annealing temperatures resulted in similar curve progressions for FD-TiBu with dropping force levels until 500 °C (5 min) and 450 °C (10 min), followed by remaining force levels and a severe force peak at 550 °C (5 min) and 500 °C (10 min) prior to loss of superelasticity. FD-TiLF was sensitive to heat using 5 and 10 min annealing temperatures with severe force loss at 550 °C and, overall, greater force loss compared to 1 min annealing temperatures. FD-TiSe wires were insensitive to heat up to 550 °C regardless of the annealing temperature. A loss of superelasticity was found for FD-TiBuat 750 °C (1 min), 650 °C (5 min) and 600 °C (10 min), as well as for FD-TiLF/-TiSe at 700 °C (1 min) and 550 °C (5 and 10 min) followed by a continuous decrease of force.

With regard to GAC wires, the following mean forces for untreated GAC-NS1 and -NS2 were found: F_loading = 5.73 N/5.53 N and F_unloading = 1.15 N/1.13 N (Figure 3h,i). 1 min treated GAC-NS1 and -NS2 wires were insensitive to heat and stable in shape up to 700 °C. Similar curve progressions were found for 5 and 10 min treated GAC-NS1 wires with stable force levels until 550 °C (5 min) and 500 °C (10 min), while 5 and 10 min treated GAC-NS2 wires presented dropping force levels until 500 °C (5 min) and 450 °C (10 min). The 1, 5 and 10 min treated GAC-NS1 and -NS2 wires presented an increase in force before loss of superelastic properties at 750 °C (1 min), 650 °C (5 min) and 600 °C (10 min), followed by a decrease in force afterwards. Overall, GAC wires were characterized by the lowest force levels compared to all other investigated wires.

The Ormco-ASE wires were characterized by mean loading and unloading forces of 7.70 N and 3.78 N in an untreated condition and a loss of superelasticity at 700 °C (1 min), 600 °C (5 min) and 550 °C (10 min), followed by decreasing force levels for all annealing temperatures regarding the unloading curve and relatively stable force levels regarding the loading curve (Figure 3j). The 1 min annealing temperatures resulted only in slight force changes until loss of superelasticity, while 5 and 10 min showed force depressions at 500 °C and 450 °C, respectively.

Regarding the RMO wires, untreated RMO-FLI27 and -FLI35 displayed the following mean force levels: F_loading = 5.34 N/4.86 N and F_unloading = 2.38 N/1.96 N (Figure 3k,l). RMO-FLI27 and -FLI35 presented relatively stable force levels for 1 min annealing temperatures up to 650 °C and 700 °C, followed by a severe decrease in force. Considering 5 and 10 min annealing temperatures of RMO-FLI27 and -FLI35, both curves depicted stable force levels until 500 °C and 450 °C, respectively, followed by a severe force loss. This force drop resulted in a complete loss of force at 600° C and 550 °C regarding the unloading curve while, in contrast, the loading curve presented force depressions at 650 °C and 600 °C, respectively, followed by a pronounced force increase. Loss of superelastic properties was found for RMO-FLI27 at 650 °C (1 min), 550 °C (5 min) and 450 °C (10 min) and RMO-FLI35 at 700 °C (1 min), 500 °C (5 min) and 450 °C (10 min).

With respect to the 3M Unitek wires, 3M-NC and -NSE depicted the following median force levels in an untreated state: F_loading = 9.06 N/8.45 N and F_unloading = 5.57 N/4.89 N (Figure 3m,n). The 3M-NC presented stable force levels followed by a continuous, unwavering drop in force from 500 °C (1 min), 400 °C (5 min) and 300 °C (10 min) onwards. The 3M-NSE was insensitive to heat and stable in shape, presenting a stable force plateau until loss of superelastic properties, and showed almost no force reduction regarding longer annealing durations for all annealing temperatures regarding the loading curve. A drop in force levels was detected at 550 °C (10 min), 600 °C (5 min) and 700 °C (10 min). A loss of superelasticity was found for 3M-NC at 500 °C (1 min), at 400 °C (5 min) and 300 °C (10 min), as well as for 3M-NSE at 700 °C (1 min), at 600 °C (5 min) and 500 °C (10 min) for 3M-NSE.

### 3.3. Detection of Favourable Working Ranges and Total Scores Achieved in the Three-Point Bending Score

**Figure 4:**
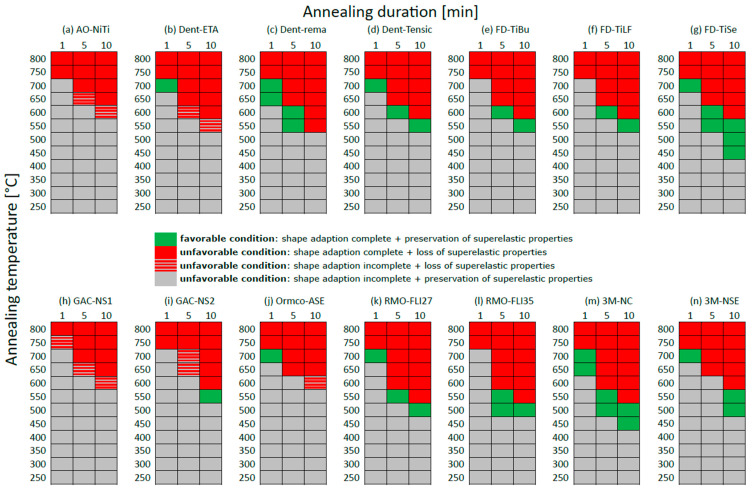
A graphical representation of the favourable and unfavourable working range (according to the results of the angle and three-point bending tests) is given for each annealing time and temperature and for each wire. A colour scheme is used to represent favourable and unfavourable working ranges.

To summarise the results of the angle and three-point bending test, so-called favourable and unfavourable working ranges were defined for each wire (Figure 4).

A favourable working range of a wire indicates that the applied annealing duration and annealing temperature presented:

(1) Complete shape acceptance (β = 180° ± 3°) of the examined wire in the angle test (Figure 2);
  AND
(2) No loss of superelastic properties of the examined wire in the three-point bending test (Figure 3).

Favourable working ranges are presented in green (Figure 4). Furthermore, working ranges were regarded as “small” if only one combination of annealing duration and temperature was found, as “user friendly” if a working range was found for at least two annealing durations with defined temperature ranges and as “most user-friendly” if a working range was found for each annealing duration (1, 5 and 10 min) with a defined annealing temperature.

Unfavourable working ranges of a wire indicate that the applied annealing duration and annealing temperature resulted either in:

- Complete shape acceptance (β = 180° ± 3°) of the examined wire in the angle test (Figure 2) AND loss of superelastic properties of the examined wire in the three-point bending test (Figure 3), which is presented in red (Figure 4);

OR

- Incomplete shape acceptance (β < 180° ± 3°) of the examined wire in the angle test (Figure 2) AND loss of superelastic properties of the examined wire in the three-point bending test (Figure 3), which is presented in shaded red (Figure 4);

OR

- Incomplete shape acceptance (β < 180° ± 3°) of the examined wire in the angle test (Figure 2) AND no loss of superelastic properties of the examined wire in the three-point bending test (Figure 3), which is presented in grey (Figure 4).

No applicable working range was found for AO-NiTi and GAC-NS1. Some wires only exhibited a very small working range for one defined annealing duration–annealing temperature combination: Dent-ETA, GAC-NS2 and Ormco-ASE. The wires Dent-rema, FD-TiBu, FD-TiLF, RMO-FLI35 and 3M-NSE presented favourable user-friendly working ranges, however, not for every annealing duration. The wires Dent-Tensic, FD-TiSe, RMO-FLI27 and 3M-NC were characterised by a favourable working range for each annealing duration with a defined annealing temperature (range) and were most user-friendly. The evaluation of the size of the working range over all three annealing times showed a clear overall superiority of FD-TiSe and 3M-NC.

Furthermore, a new evaluation score (Table 2) was designed to evaluate the quality of the complex force–deflection curves (1.5 mm) and to summarise findings of the three-point bending test (Figure 3) numerically (Table 3). Regarding the scores of the individual wires, it is noticeable that none of the tested wires sets could reach the maximum achievable total score of 3 × 38 for each annealing duration (in total 114 for all annealing temperatures) (Table 3). Scores achieved for the 1 min/5 min/10 min annealing duration, which did not present a defined working range for the examined wire at the tested annealing duration, were added to the total score. The total score of wires without an existing working range for all three annealing temperatures were considered as “not applicable” (Table 3, red). Overall, the scores for the 5/10 min annealing durations were lower than those for 1 min annealing accompanied by the least significant effect on the material properties.

Notably, wires with the most useful working ranges in the angle test did not necessarily show the best scores in the bending test (Table 3), e.g., GAC-NS1 obtained high scores for each annealing temperature but showed a non-existent working range in the angle test and, therefore, the total score of GAC-NS1 was regarded as “not applicable” (Table 3, red). A temperature insensitive wire should ideally achieve similar scores at all three annealing temperatures, which was found for AO-NiTi, Dent-rema and FD-TiSe. However, wires with similar achieved scores in the three-point bending test did not always present favourable working ranges for each annealing temperature, e.g., AO-NiTi presented similar scores for each annealing temperature, but was characterized by a non-existent working range. Dent-rema showed similar scores for each annealing temperature, however, the 10 min annealing temperature was not added to the total score since no favourable working range was found for Dent-rema at 10 min annealing duration.

Considering the results of the angle and three-point bending test using the working range and the total score achieved in parallel, Dent-Tensic, FD-TiSe, FD-TiLF, RMO-FLI27 and 3M-NC presented the most user-friendly working ranges for all annealing temperatures and, overall, had the highest total scores (Table 3, green).

## 4. Discussion

In the present study, the influences of thermal shape-setting on the material properties of 14 rectangular superelastic NiTi archwires with uniform arch dimensions (0.018 × 0.025 inch) were tested using a ceramic heating furnace and a shape setting mould, ensuring reproducible test conditions and enabling valid conclusions.

A limitation of the study is that the three-point bending test is only suitable to a limited extent for superelastic NiTi, since the test is based on conventional elasto-mechanics assuming linear-elastic behaviour. Due to the complexity of the mechanical models, simplified use must therefore be made of linear elasticity theory in the context of this work. Furthermore, the three-point bending test was also applied in previous studies investigating mechanical properties of NiTi archwires [5]. A further limitation is that the herein defined evaluation scale (“score”) for the three-point bending test involved some unavoidable subjectivity. Consequently, the evaluation scale represents only a tendency for the suitability of each wire.

The aim of the heat treatment in the furnace was to ensure constant experimental conditions for the heat treatment process, which is not the case if the electrical heat source (“Memory-Maker”) is applied. So far, previous studies investigated the impact of heat treatment on three rectangular superelastic archwires (0.016 × 0.022 inch) [7] and 10 round superelastic NiTi (0.016 inch) archwires [11]. However, those studies only investigated small temperature ranges of 550–650 °C with increments of 50 °C [7] or 400 and 600 °C, [11] as well as very short (2 or 5 s) [7] or very long (1 h) heating durations [11]. In addition, heat treatment of commercial NiTi archwires was used in the so-called ‘ageing method’ by applying an Ni-rich precipitate into the alloy matrix and treatment of those archwires with different temperatures from 370 °C to 520 °C (increment of 30 °C) for 15 min [5].

Our findings detected that complete bending occurred after 1 min annealing duration only at relatively high temperatures, whereas it was observed at much lower temperatures during 5/10 min annealing. This indicates that, under the given conditions, the warming of the tool used was incomplete after the short duration of 1 min. As the thermal conductivity of the brass mould was very good, the mould itself had a certain thermal mass, which in return affected the actual chamber temperature once placed inside the furnace. It seems that the short treatment duration of only 1 min was not long enough to compensate for the temperature drop caused by the thermal mass of the mould. Notably, treatment of three NiTi archwires using even shorter annealing durations of 1 to 5 min also resulted in incomplete shape adaptation (sweep) when applying lower annealing temperatures [7]. This also explains the steep changes and the variability of the curves shown in Figure 3, where in almost every diagram the curves for 1 min annealing time show effects at higher temperatures, while 10 min annealing time shows material reactions at much lower temperatures. The 5 min annealed samples are always in between. For some samples (Figure 3b,g,k,l), the temperatures above 650 °C show a certain increase in upper plateau force, indicating that the recrystallisation of the material has already at least partly taken place. Other samples (Figure 3b,d,e,h) show increasing lower plateau forces at intermediate temperatures. This may be attributed to the occurrence of an R-phase transformation, which is known as a rhombohedrally distorted Austenite along the [111]-direction of the B2 crystal structure [12]. The R-phase is stabilized by annealing processes at lower temperatures and causes some irregular functional properties, such as smaller mechanical hysteresis in force–deflection tests.

Furthermore, it was noticeable that often only a small temperature working range existed in which, on the one hand, the desired shape setting was complete while, on the other hand, the superelastic properties of the wire were preserved. Accordingly, only distinct combinations of annealing temperature and annealing duration led to the desired results in a previous study, e.g., 600 °C for 1 to 5 min was most promising for the measured archwires [7]. In this study, FD-TiSe, Dent-Tensic, RMO-FLI27 and 3M-NC proved to be the most user-friendly, as they exhibited for each annealing duration a distinct range of suitable annealing temperatures. FD-TiSe and 3M-NC also presented the broadest working range, indicating that these wires have the most insensitive properties for thermal treatment.

Some wires showed only narrow working ranges of 50 °C for one defined annealing duration, e.g., Dent-ETA, Ormco-ASE or GAC-NS2, which indicates that thermal programming of these wires is very sensitive. Two wires presented no working range (AO-NiTi, GAC-NS1) with a loss of superelastic properties at complete shape acceptance and even before complete shaping, which would make these wires orthodontically unusable in cases where thermal shape adaptation is needed due to their sensitivity to heat treatment of any kind.

An investigation of microstructural changes due to heat treatments was not part of the current research, mainly because the correlation between clinically relevant functional properties and microstructural changes of Ni-rich NiTi alloys have already been addressed in separate research [13,14]. However, it is well known from the literature that heat treatments of any kind affect both the structural and functional properties of the material. The following changes may occur in Ni-rich alloys during heat treatment and affect the relevant functional properties in a complex manner:

- Changes in transformation sequence (occurrence of R-phase after lower heat treatment temperatures and higher amounts of cold work applied during wire manufacturing).
- Growth of Ni-rich Ni_4_Ti_3_ precipitates, leading to an increase of transformation temperatures during heat treatment due to loss of Ni from the matrix. The process is diffusion controlled and depends on the dislocation density because dislocations are nucleation sites for precipitation during subsequent annealing.
- Recovery and recrystallisation during heat treatments at higher temperatures, leading to a softening of the material and reducing the resistance against plastic deformation, as well as the nucleation sites for further precipitation.

These effects were occurring simultaneously in Ni-rich NiTi alloys and can be visualized in Time–Temperature–Transformation (T–T–T) diagrams [14].

Due to its good reproducibility, the three-point bending test represents a standard method for recording the mechanical properties of orthodontic wires and providing information about material characteristics, e.g., force level and position of the superelastic plateau. It was also used in recent studies investigating the mechanical properties of NiTi archwires after heat treatment [5,15,16]. One study chose the three-point bending test to compare direct electrical resistance heat treatment or cold forming to bend defined offsets or angles into rectangular NiTi archwires (0.016 × 0.022 inch). Notably, this study presented no differences regarding the mechanical properties and A_f_ temperatures when comparing both methods, however, force levels decreased when the distance of the bending was enhanced [15]. Another study using the three-point bending test after application of direct electrical heat treatment after 1.5 mm deflection of superelastic and heat-activated NiTi archwires detected that the plateau in the force–deflection curve of both wires was increased and that longer annealing duration led to a loss of superelastic properties [16]. In our study, the three-point bending test provided the expected curve shape of a superelastic wire for all untreated samples. Special attention was paid to the plateau of the unloading curve as it is used for therapeutic purposes. Comparing the 1, 5 and 10 min curves, it was noticeable that at low annealing temperatures of ~350 °C, all three curves showed a similar force level. Only at medium temperatures of 350–550 °C did the 5 and 10 min curves present significantly lower force values. Longer annealing times were characterised by loss of superelastic behaviour (=plastic state) already at lower temperatures. Taken together, the tested wires could be subdivided into two groups:

(1) Wires which exhibited relatively stable force values F_loading (1.5 mm) until the transition to the plastic state and only reacted with a drop in force in the plastic state:
  AO-NiTi, Dent-rema, FD-TiLF/TiSe, Ormco-ASE and wires from RMO and 3M Unitek.
  Due to their stable force levels over a wide heat treatment range, these wires were regarded as user-friendly for thermal shape-setting.
(2) Wires with force peaks followed by force drops before transitioning to the plastic state:
  Dent-ETA/-Tensic, FD-TiBu and GAC wires.
  These wires were regarded as problematic for thermal shape-setting since the observed force peaks after heat treatment could lead to clinical side effects, e.g., the risk for orthodontically induced inflammatory root resorption was shown to be increased when heavy orthodontic forces are applied [17].

## 5. Conclusions

In summary, it can be concluded from this study that good material and processing knowledge should be available for thermal shape setting in order to avoid the loss of properties as far as possible, since significant changes in the material properties were found, e.g., loss of superelastic properties when distinct annealing temperatures and durations were exceeded. Notably, complete shape adaptation without loss of superelasticity can only be carried out in a small working range for most of the wires, while some wires presented greater working ranges (FD-TiSe, 3M-NC) and some presented a non-existent working range (AO-NiTi, GAC-NS1). A newly designed evaluation score to summarise mechanical properties found in the three-point bending test detected the highest scores for the wires Dent-Tensic, FD-TiSe and RMO-FLI273M-NC. Taken together, these wires were considered to be the most user-friendly, since the desired shape can be adjusted into these wires within a favourable working range and without assuming a loss of the superelastic properties because they exhibited relatively constant force levels at all three temperatures. In the long term, this study is expected to facilitate thermal shape adaptation and provide a possible basis for further developments of NiTi thermal shape setting.

## Figures and Tables

**Table 1:** Orthodontic superelastic NiTi wires tested in this study.

Manufacturer	Product Name	LOT N°	Abbreviation
American Orthodontics	Nickel-Titanium	857-7451	AO-NiTi
Dentaurum	Equire Thermo-Active	766-815-00	Dent-ETA
	rematitan “Lite”	766-077-00	Dent-rema
	Tensic	766-717-00	Dent-Tensic
Forestadent	Titanol-Budget	206-2146	FD-TiBu
	Titanol Low Force	280-2145	FD-TiLF
	Titanol Superelastic	204-2146	FD-TiSe
GAC	Neo Sentalloy	02-526-652	GAC-NS1
	Neo Sentalloy	02-523-653	GAC-NS2
Ormco	Align SE200 LM NiTi	227-3116	Ormco-ASE
Rocky Mountain Orthodontics	FLI CuNiTi27	WCN 7837	RMO-FLI27
	FLI CuNiTi35	WCN 7856	RMO-FLI35
3M Unitek	Nitinol Classic	4297-714	3M-NC
	Nitinol Superelastic	4297-814	3M-NSE

**Table 2:** Criteria for the evaluation of superelastic wires using an evaluation score.

Criteria	Height of the Upper Plateau [N]					Stability of the Lower Plateau during Heat Treatment, Until [°C]											
Value	5	6	7	8	≥9	700	650	600	550	500	450	400	350	300	250		
Index	4	3	2	1	0	10	9	8	7	6	5	4	3	2	1		
Criteria	Height of the Lower Plateau [N]					Slope With the Change of the Lower Plateau ∆F/∆T [N/100 °C]											
Value	1	2	3	4	≥5	0.5	1	1.5	2	2.5	3.0	3.5	4.0	4.5	5.0	5.5	6.0
Index	4	3	2	1	0	10	9	8	7	6	5	4	3	2	1	0	−1
Criteria	Evaluation of the Overall Force–Deflection Curve Shape																
Value	Monotonous, small deflections					Monotonous, significant deflections		Not monotonous, small deflections		Not monotonous, medium deflections		Not monotonous, clear deflections		Chaotic, not predictable			
Index	10					7		5		3		2		1			

**Table 3:** Favourable working ranges, scores achieved and total scores of all wires regarding the angle and three-point bending test.

Abbreviation	Favourable Working Ranges *		Total Temperature Working Range	Score Achieved			Total Score	Total
Manufacturer-Product	Annealing Duration	Annealing Temperature		1 min	5 min	10 min		Score (%)
AO-NiTi	None	None	Non-existent	25	25	25	N.A.	N.A.
Dent-ETA	1 min	700 °C	Small range: for 1 min annealing duration	24	16	13	24	21.1
Dent-rema	1 min	650–700 °C	User friendly:	23	25	23	48	42.1
	5 min	550–600° C	for 5 and 10 min annealing duration					
Dent-Tensic	1 min	700 °C	Most user friendly:	27	19	18	64	56.1
	5 min	600 °C	for all annealing durations					
	10 min	550 °C						
FD-TiBu	5 min	600 °C	User friendly:	26	21	17	38	33.3
	10 min	550 °C	for 5 and 10 min annealing duration					
FD-TiLF	5 min	600 °C	User friendly:	33	25	24	49	43.0
	10 min	550 °C	for 5 and 10 min annealing duration					
FD-TiSe	1 min	700 °C	Most user friendly:	23	24	24	71	62.3
	5 min	550–600° C	for all annealing durations					
	10 min	450–550 °C	greatest temperature working ranges					
GAC-NS1	None	None	Non-existent	34	28	28	N.A.	N.A.
GAC-NS2	10 min	550 °C	Small range: for 10 min annealing duration	33	25	23	23	20.2
Ormco-ASE	1 min	700 °C	Small range: for 1 min annealing duration	27	25	22	27	23.7
RMO-FLI27	1 min	700 °C	Most user friendly:	27	20	19	66	57.8
	5 min	550 °C	for all annealing durations					
	10 min	500 °C						
RMO-FLI35	5 min	500–550 °C	User friendly:	34	21	21	42	36.8
	10 min	500 °C	for 5 and 10 min annealing duration					
3M-NC	1 min	650–700 °C	Most user friendly:	22	18	18	58	50.9
	5 min	500–550 °C	for all annealing durations					
	10 min	450 °C	greatest temperature working ranges					
3M-NSe	1 min	700 °C	User friendly:	23	21	26	49	43.0
	10 min	500–550°C	for 1 and 10 min annealing duration					

* favourable working range = shape acceptance (β = 180° ± 3°) in the angle test without loss of superelastic properties in the three-point bending test of the examined wire; N.A. = not applicable (total score is not applicable as no favourable working range was found for the examined wire); colour scheme: green = favourable working range for each annealing duration; red = unfavourable working range for the tested annealing duration.

## Data Availability

The data presented in this study are available on request from the corresponding author. The data are not publicly available.
